# Nonlinear Optical Detection of β2-Microglobulin Exploiting Graphene-Enhanced Terahertz Third-Harmonic Generation

**DOI:** 10.3390/s26216779

**Published:** 2026-10-26

**Authors:** Ghassem Baridi, Arslan Liaquat, Federico Rapuzzi, Herath Mudiyanselage Kasun Gayanga Anuradha Herath, Maria Celeste Maschio, El Hadj Abidi, Vito Clericò, Yahya Moubarak Meziani, Mario Amado, Enrique Diez, Stefano Corni, Giorgia Brancolini, Luigi Rovati, Francesco Rossella

**Affiliations:** 1 Department of Engineering “Enzo Ferrari”, University of Modena and Reggio Emilia, Via P. Vivarelli, 10, 41125 Modena, Italy; luigi.rovati@unimore.it; 2 Department of Physics, Computer Science and Mathematics, University of Modena e Reggio Emilia, Via Campi 213/a, 41125 Modena, Italyf.rossella@unimore.it (F.R.); 3 Istituto Nanoscienze—CNR, S3, Via G. Campi 213/A, 41122 Modena, Italygiorgia.brancolini@nano.cnr.it (G.B.); 4 Department of Applied Physics, University of Salamanca, 37008 Salamanca, Spain; 5 Nanotechnology Group, USAL–Nanolab, Departamento de Física Fundamental, University of Salamanca, Plaza de la Merced, Edificio Trilingüe, 37008 Salamanca, Spain

**Keywords:** third-harmonic generation, biosensor, electric double layer, electrolyte gating, graphene

## Abstract

β2-Microglobulin (β2M) is a crucial biomarker associated with kidney dysfunction, dialysis-related amyloidosis, and inflammatory disorders, encouraging the development of sensitive sensing platforms. Here, we suggest a nonlinear terahertz (THz) sensing platform based on graphene-enhanced third-harmonic generation (THG) for β2M detection. In contrast with conventional graphene plasmonic sensors that depend on linear resonance shifts, the proposed approach investigates the strong dependence of graphene’s nonlinear optical response on its Fermi energy. Adsorption of charged β2M molecules changes graphene’s carrier concentration and Fermi energy, resulting in a detectable signal in the THG response. The sensor includes periodically patterned graphene microribbons integrated with a gold substrate with a thin Kerr nonlinear dielectric spacer, forming a resonant metasurface with strong THz field confinement. The combined impacts of graphene plasmonic resonances and cavity-enhanced field localization significantly increase THG at relatively low incident intensities. The impact of graphene Fermi energy, dielectric thickness, and excitation intensity on the sensing performance are systematically investigated. The simulation results predict concentration-dependent shifts in the THG spectral response and intensity, with increasing β2M concentration producing a progressive shift toward higher frequencies. The simulations predict β2M detection at concentrations as low as 0.0001 g/L, achieving a sensitivity of 971 THz·L/g and a figure of merit (FOM) of 1706 L/g. These theoretical results represent the potential of graphene-enhanced THG metasurfaces for sensitive, tunable, and compact THz biosensing and biomedical diagnostics.

## 1. Introduction

Biosensors have emerged as important analytical devices for early disease diagnosis [1], personalized medicine [2] and biomolecular analysis [3]. Based on the International Union of Pure and Applied Chemistry (IUPAC), a biosensor includes a detectable recognition element with a transducer to change biochemical interactions into measurable signals [4]. Between variety sensing platforms, optical biosensors have become attractive candidates because of high sensitivity, rapid response, and label-free detection capabilities [5]. They have been considerably used for the detecting of biomolecules such as proteins [6], nucleic acids [7], antibodies [8], and disease biomarkers [9], enabling applications in medical diagnostics [10], environmental monitoring [11] and biotechnology [12].

Progress in nanophotonics and two-dimensional (2D) materials have opened new gates for nonlinear optical biosensing, while nonlinear optical effects have become the basis of a wide domain of modern photonic technologies, including laser frequency conversion [13], ultrashort pulse generation [14], all-optical signal processing [15], and ultrafast optical switching [16]. These impacts are also enhanced in nanoscale photonic devices, where strong light–matter interactions can remarkably increase optical responses. Among two-dimensional materials, graphene has achieved remarkable attention because of its exceptionally high third-order nonlinear optical susceptibility, making it an excellent candidate for third-harmonic generation (THG)-based sensing [17]. In dispute with the traditional linear sensing regime based on alters in reflectance or resonance conditions, THG-based biosensors apply the nonlinear interaction between intense electromagnetic fields and graphene, improving enhanced sensitivity to local refractive-index variations generated by biomolecular binding events [18]. These special nonlinear properties make graphene a promising material for the achievement of highly sensitive and compact optical biosensors [19,20]. Generally, nonlinear optics evaluate how materials interact with high-intensity light where the polarization no longer scales linearly with the electric field. Under such circumstances, traditional linear phenomena—such as reflection, refraction, and scattering, which appear mainly from photon–electron interactions—occur alongside much weaker nonlinear contributions. These nonlinear processes begin with effective photon–photon interactions and typically appear only due to strong electromagnetic fields. Meanwhile, in perfectly engineered materials or structures that increase local field strength, nonlinear responses can be boosted, supporting higher efficiency and stronger functional effects [21,22]. The field of nonlinear optics is considered to have begun with the observation of second-harmonic generation (SHG) by Franken and colleagues in 1961, shortly after Maiman showed the first functioning laser in 1960. Optical impact is back to nonlinear when a material’s response to an electromagnetic field no longer scales relatively with the field’s strength. A most famous case is SHG, which appears from components of the atomic polarization that shift with the square of the applied field. In result, the output at twice the fundamental frequency increases approximately with the square of the incident laser intensity [23].

Between variety 2D material, graphene demonstrates considerably high nonlinear optical behavior when exposed to an electromagnetic field. Because of the linear energy dispersion of its Dirac fermions, graphene shows considerable nonlinear responses, especially in the terahertz and microwave range. Its nonlinear optical characters have been extensively investigated by techniques such as four-wave mixing, which show that graphene exhibits a very large third-order optical susceptibility that changes only somewhat with wavelength in the infrared range. Besides that, this third-order nonlinearity can be controlled electrically, allowing tunability of the optical response.

Traditionally, the nonlinear behavior of an optical medium generates from its optical constants because of the effect of intense electromagnetic fields [24]. Graphene’s crystal structure has a D6h point group, which is considered as a centrosymmetric two-dimensional material. Because of this inversion symmetry, all second-order nonlinear optical processes are forbidden in pristine graphene under the electric-dipole approximation. Consequently, phenomena such as SHG, second-order susceptibility and connected second-order nonlinear conductivities do not exist in pristine monolayer graphene [25]. In contrast, graphene shows considerable high third-order nonlinear optical behavior. Specifically, its third-harmonic generation (THG) efficiency is enough strong in compare with various conventional bulk materials, due to its linear energy dispersion, high carrier mobility, and strong light–matter interaction at atomic scale. These third-order effects—including THG, Kerr nonlinearity, and four-wave mixing—enable graphene to act as a strong platform for nonlinear photonic modern devices [25]. In the THG phenomenon frequency with 3*ω* generated by an applied field at frequency *ω*, three photons of frequency *ω* are destroyed and one photon with frequency 3*ω* is created in this process [24,25,26,27].

Graphene third-harmonic generation (THG) has been investigated experimentally and theoretically. Hong et al. demonstrated optical THG in graphene [17], while Rostami and Polini [18] and Cheng et al. [27] developed theoretical descriptions of its third-order optical response. Numerical studies have subsequently investigated enhancement of nonlinear conversion using plasmonic–graphene metasurfaces [28], graphene-assisted meta-grating structures [29], and graphene microribbon arrays [30,31]. These works provide the foundation for graphene-based nonlinear frequency conversion, but the cited studies do not couple protein adsorption and electrolyte-induced electrostatic gating to a THG biosensing readout.

Our previous computational study of β_2_-microglobulin detection using electrolyte-gated graphene investigated a linear surface-plasmon-resonance response [32]. The present work extends this framework to a nonlinear THG readout by linking molecular simulations of protein adsorption, electric-double-layer-induced changes in graphene’s carrier density and Fermi energy, and the resulting third-order nonlinear optical response. Its specific contribution is the prediction of concentration-dependent THG spectral changes within this multiscale framework. The proposed sensing modality and its predicted performance require experimental validation.

In this manuscript, we demonstrate a combined atomistic and optical modeling framework for investigating the sensing of β2-microglobulin (β2-m) through third-harmonic generation (THG) in graphene-based sensing platforms. The computational approach combines SDA version 7.2.2 software, GROMACS, and COMSOL Multiphysics version 6.2 to capture the molecular interactions and their corresponding optical responses across multiple length scales. Emphasis is placed on the wild-type β2-m monomer and its interaction with the graphene surface during the early stages of adsorption. The primary objective is to examine how adsorption-induced conformational changes, charge redistribution, and interfacial physicochemical interactions influence the optical characteristics of the device. By establishing a direct connection between protein-level molecular patterns and theoretically measurable third-harmonic signals, the framework provides valuable insights into the mechanisms governing graphene-based biosensing performance. Here, the sensing response is evaluated from the resonance-frequency shift of the third-harmonic generation (THG) spectrum, with the resonance position determined from the THG response under different analyte concentrations. Accordingly, the reported sensitivity and figure of merit characterize the nonlinear THG-based readout of the proposed graphene plasmonic structure. The present study focuses on identifying a plasmonic configuration that maximizes the THG resonance shift under identical operating and analyte conditions. A direct quantitative comparison between linear and THG readouts using the same structure, analyte model, and noise assumptions is beyond the scope of the present work and will be considered in future studies.

In this work, graphene has an initial Fermi energy of approximately 0.3 eV, with the Dirac point around 2 V. The negatively charged β-2M (−2e) is treated as an external electrostatic charge rather than as direct charge transfer to graphene. Its presence in the electrolyte modifies the electric double layer (EDL), resulting in a change in graphene’s carrier density and Fermi energy. Thus, the key mechanism considered here is EDL-induced electrostatic gating.

## 2. Theory and Design 

**Figure 1:**
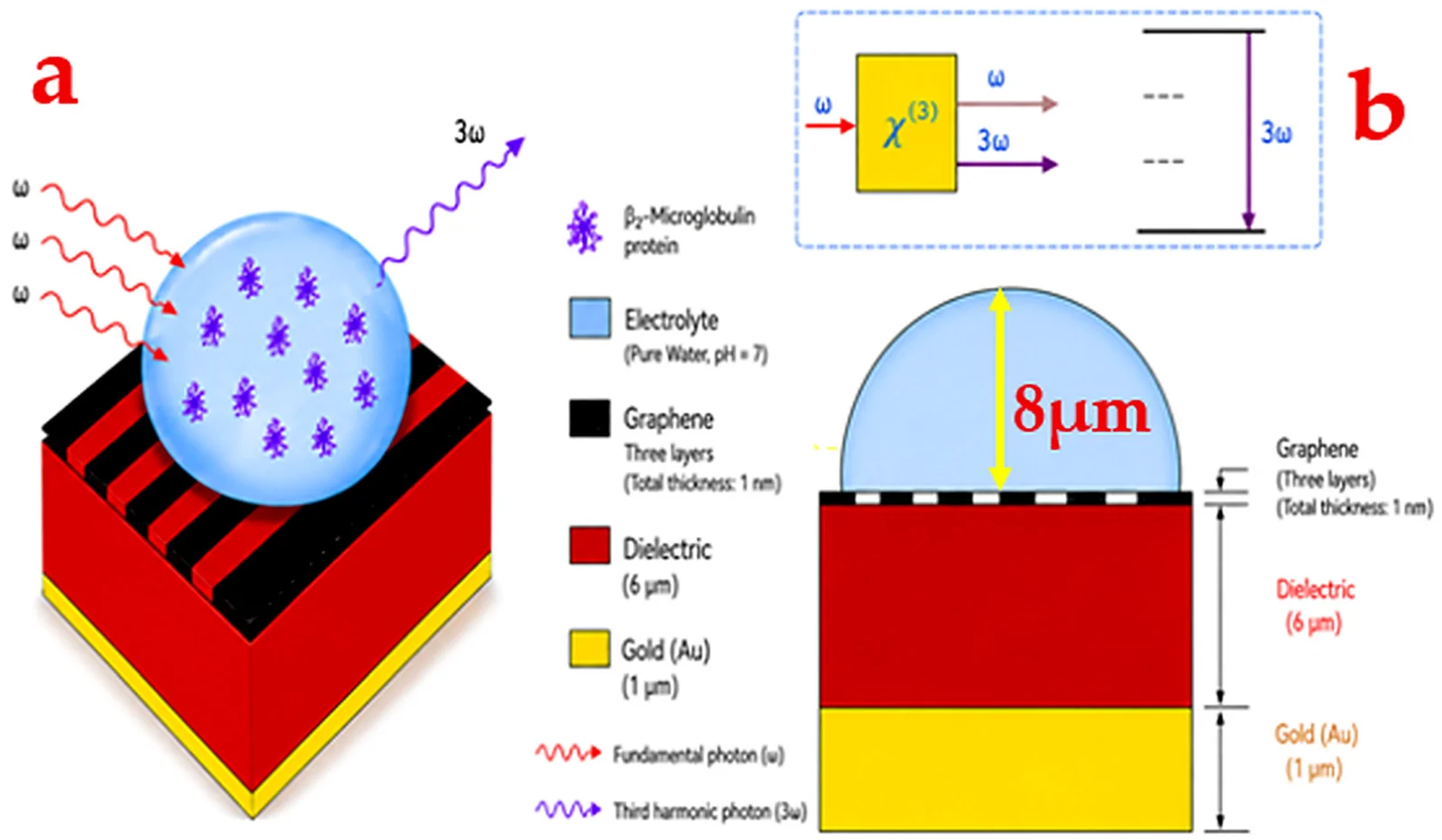
Schematic of the proposed nonlinear graphene metasurface and interaction process: (**a**) 3D view of graphene nanoribbons on a dielectric layer over a gold substrate; (**b**) 2D cross-sectional view (graphene in black, dielectric in red, gold in yellow); inset: third-harmonic generation schematic mechanism.

Figure 1 illustrates the geometry of the proposed nonlinear graphene metasurface. Metasurfaces are two-dimensional metamaterials whose subwavelength-scale unit cells (meta-atoms) can be geometrically engineered to control the phase, amplitude, and polarization of light, providing high-performance optical devices and modern manipulation of light propagation [33]. Their design properties consist of an array of graphene nanoribbons positioned on a dielectric, which is homogeneously and periodically arranged to top a gold substrate. The dielectric material has a refractive index of 2.1, and the gold substrate maintains a constant thickness of 1 μm. To minimize reflection and absorption of scattered waves at the boundaries, perfectly matched layers (PMLs) are used at both the top and bottom of the structure. The overall schematic of this setup is demonstrated in Figure 1. The computational domain is discretized employing a highly refined mesh.

Graphene’s optical properties come from its specific two-dimensional band structure and are controlled by intraband and interband electronic transitions [28,29,30]. These transitions allow broadband optical conductivity, ultrafast carrier dynamics, and robust light–matter interaction, making graphene perfect candidate for photonic and optoelectronic applications [28,29,30]. The linear optical conductivity related to intraband transitions is generally described by the Drude dispersion model, described as [34,35]

(1) $$\sigma_{\mathrm{Gra}}\approx \frac{-\mathrm{iD}}{\pi (\omega -\frac{i}{\tau })}$$

where

(2) $$D=\frac{e^{2}E_{f}}{\hbar^{2}}$$

is the electron relaxation time that accounts for the optical loss, *e* is the electron charge, angular frequency, and ħ is the reduced Planck constant [34,35]. The linear conductivity of graphene depends on the wave frequency, Fermi energy, and electron relaxation time, which is affected by temperature, external fields, sample quality, and substrate material, according to

(3) $$\tau =\frac{\mu E_{f}}{ev_{f}^{2}}$$

where *μ* is the mobility of the graphene sample and *v_f_* is the Fermi velocity 1 ∗ 10^6^ ms^−1^. It has been reported that the mobility of graphene may exceed 20 m^2^V^−1^s^−1^ at room temperature and the amount of *τ* is in the picosecond range [24]. Due to its 2D nature, graphene has a sub-nanometer thickness, which can be ignored in numerical computation and treated as a surface current. The linear element of this surface current is written as

*j*(*ω*) = *σ_gr_*(*ω*)*E*(*ω*) (4)

where *E*(*ω*) is the electric field along the x-direction on the surface [24]. The nonlinear optical response of graphene, typically, can be written in terms of the nonlinear surface conductivity tensor ($\sigma_{s}^{\left(3\right)}$). It is described as below [24,25,26]:

(5) $$\sigma_{\mathrm{gr}}^{\left(3\right)}=\;\frac{i\sigma_{0}{\left(\hbar v_{f}e\right)}^{2}}{48\pi {\left(\hbar \omega \right)}^{4}}T(\frac{\hbar \omega }{2E_{f}})$$

*T*(*x*) = 17*G*(*x*) − 64*G*(2*x*) + 45(3*x*) (6)

and

(7) $$G\left(x\right)=\left|\left(\frac{1+x}{1-x}\right)\right|+\mathrm{i\pi \theta}(\left|x\right|-1)$$

With *θ(z)* as the Heaviside step function, the third-order conductivity is controlled by its imaginary part, while scattering and thermal effects are not considered [24,25,26,27]. In third-harmonic generation (THG), three photons at the fundamental frequency (*ω_FF_*) combined to generate one photon at *ω_TH_* = 3*ω_FF_*, satisfying energy conservation [24,26]. The resulting nonlinear surface current is demonstrated by [24,25,26,27,28,29]

(8) $$j^{3}(\omega )=N\sigma_{\mathrm{gr}}^{\left(1\right)}\left(3\omega \right)E_{\mathrm{TH}}+\sigma_{\mathrm{gr}}^{\left(3\right)}\left(3\omega \right){\left(E\left(\omega \right)\right)}^{3}$$

where N is the number of graphene layer. In our simulation, we applied three layers of graphene. Third-order nonlinear effects in ultra-thin materials, such as graphene, are naturally weak, which requires high input intensities to generate observable nonlinear responses. Even with these high inputs, the resulting nonlinear signals are often low, limiting the overall efficiency of nanoscale optical devices [33,34,35]. In the nanoscale, enhancing nonlinear impacts are vitally related to the high confinement of the optical field and its spatial overlap with the material. Plasmonic and metasurface structures can offer this confinement by concentrating on light into deep subwavelength volumes, thus increasing light–matter interactions. Thus, both theoretical and experimental efforts in nonlinear nanophotonics have been focused on designing structures that maximize near-field enhancement and mode overlap, providing higher-performance third-order nonlinear processes in ultra-thin materials [36].

Plasmonic is the study and manipulation of light at the nanoscale, focusing on how electromagnetic waves interact with collective electron oscillations in metals, known as surface plasmons [37]. In application optical devices, metallic plasmons are often limited by ohmic losses and the intrinsically fixed features of metals, which cause energy dissipation as heat and limit the propagation length of plasmonic waves. Moreover, the superficial penetration of electromagnetic waves into metals means that the optical response of bulk metals is mainly determined by weak light–matter interactions near the metal–dielectric interface. At the nanoscale, in contrast, surface plasmon polaritons (SPPs) can enhance dramatically light–matter interactions, permitting enhanced nonlinear optical phenomena [38]. Graphene plasmonic has been wildly investigated due to its capability to control electromagnetic energy at scales below the wavelength of light. A key property of graphene is its strong near-confinement allowed by surface plasmon polaritons (SPPs). In addition to metals, graphene’s optical response can be actively controlled by using gate voltage or chemical doping, making it a highly flexible material for optoelectronic applications. Its two-dimensional structure allows SPP-induced field enhancement to exceed what is generally possible in conventional metallic systems. Additionally, graphene shows relatively low losses at high carrier densities, further growing its attraction for photonic and optical devices. These characters make graphene a perfect material for advanced applications, including optical sensing, photonic switching, and communication technologies [38,39].

The main purpose of this work is to design a highly sensitive graphene-based sensor based on third-harmonic generation (THG) to sense noticeable low concentrations of biomolecules such as β_2_-microglobulin (β_2_M). β_2_M is a small (~12 kDa), non-glycosylated protein that constitutes the light chain of a major histocompatibility complex class I (MHC-I) molecules, expressed on nearly all nucleated cells [40]. It is continuously released into circulation and primarily eliminated via renal filtration, making its serum concentration a reliable indicator of kidney function [41]. In healthy individuals, β_2_M levels typically fall within 1–3 mg/L, whereas significantly elevated concentrations are associated with renal dysfunction, inflammatory conditions, and hematological malignancies [42]. In patients undergoing long duration hemodialysis patients, β_2_M can accumulate up to 20–60 mg/L, contributing to protein aggregation and dialysis-related amyloidosis [43]. Furthermore, β_2_M serves as a clinically relevant prognostic marker in disorders such as myeloma and lymphoma, where its levels correlate with disease progression and tumor burden [44].

The sensing mechanism considered in this work is based on the electrostatic coupling between β_2_M adsorption at the graphene/electrolyte interface and the electronic properties of graphene. To establish a connection across length scales, we combined atomistic molecular-dynamics simulations with continuum electrostatic calculations and electromagnetic simulations of the THG response. The resulting workflow is made of the following steps: (i) molecular characterization of β_2_M adsorption on graphene, (ii) representation of the resulting interfacial charge perturbation within the electrolyte, (iii) calculation of the corresponding electric-double-layer (EDL) response and graphene electrostatic gating, and (iv) evaluation of the resulting modification of the THG spectrum. The concentration-to-THG transduction mechanism relies on the relation between β_2_M concentration, electric-double-layer (EDL) perturbation, graphene carrier-density modulation, and the subsequent THG spectral pattern. The predicted response is affected by the assumed material character, geometrical parameters, EDL characteristics, and interfacial conditions. Uncertainties in these factors may impact the resonance shift and linewidth, and thus the computed sensitivity and figure of merit. Because this study depends on deterministic simulations, further parameter-sensitivity analyses and experimental validation are needed to assess the robustness of the suggested sensing mechanism and establish its practical performance.

**Figure 2:**
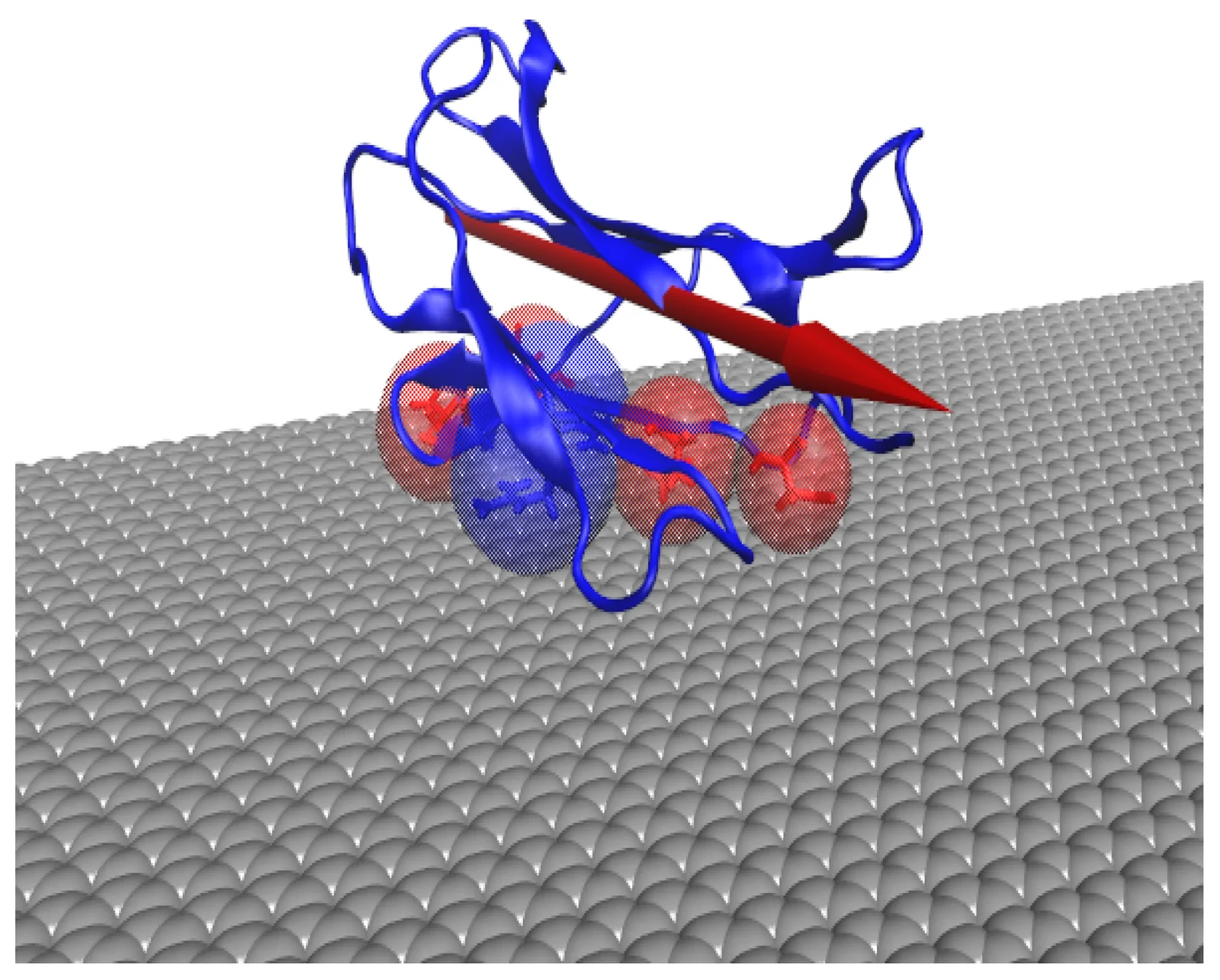
Dipole moment of 333.21 Debye of β_2_-microglobulin (β_2_M) adsorbed on graphene, calculated from 3.1 μs of Temperature Replica Exchange Molecular Dynamics (T-REMD) simulations. The results illustrate the contribution of adsorbed biomolecules to the interfacial electric double layer (EDL), beyond that of electrolyte ions alone. Positively and negatively charged residues interacting with the graphene surface at the interface are shown in blue and red (licorice representation), respectively, highlighting charge distribution at the interface relevant to local field modulation.

The molecular-scale description of β_2_M adsorption on graphene is obtained from Molecular Dynamics (MD) simulations reported in Maschio’s PhD thesis [45,46], which highlight the role of the protein in reshaping the electric double layer (EDL) at the interface. The simulations indicate that the protein preferentially adsorbs onto graphene adopting a stable orientation, where specific amino acid residues establish direct contact with the surface. The most stable orientation of the protein on graphene, obtained from enhanced sampling simulations using Temperature Replica Exchange Molecular Dynamics (T-REMD), is shown in Figure 2. These simulations, with an aggregated sampling time of 3.1 μs, were carried out using the OPLS-AA force field and the SPC/E water model within the GROMACS simulation package [47].

These results reveal that β_2_M tends to adopt a predominantly horizontal orientation relative to graphene sheet (Figure 2). Among the protein residues in contact with the surface at distances smaller than 3.5 Å, ASP34, GLU36, and GLU44 carry a negative charge, ARG45, LYS48 and ARG81 carry a positive charge, while the remaining residues are neutral. This local charge distribution contributes to the local modulation of the electrostatic potential at the interface, depending on the overall protein charge.

The adsorption of β_2_M onto graphene leads to a detectable and stable modification of the interfacial electrostatic environment, owing to its size and net charge. This, in turn, affects the surface potential and influences the sensor response [48]. The sensing mechanism assumes an aqueous electrolyte environment, consistent with physiological conditions, where β_2_M is present in solution and interacts with the graphene surface. The atomistic results provide the molecular basis for the continuum electrostatic description. In the continuum model, β_2_M is represented by an effective charged object positioned at the graphene/electrolyte interface, while retaining the dimensions and net electrostatic character identified from the atomistic simulations. For the purposes of the present model, β_2_M is approximated as a sphere with an estimated diameter of ≈3.0 nm and with a computed hydrodynamic diameter in the range of 4.5–5.0 nm, as estimated using HYDROPRO10 [46], accounting for hydration effects relative to an equivalent spherical volume. The protein diameter, derived from atomistic simulations, is incorporated as an input parameter in the COMSOL-based calculations. In this continuum Poisson–Boltzmann model, β_2_M is represented as an effective charged macroion with its total charge (−2), providing a coarse-grained description of its long-range electrostatic contribution while averaging out residue-specific charge heterogeneity at the graphene interface.

In the present research, the net charge of β_2_M is treated as an external electrostatic perturbation rather than as direct charge transfer to graphene. This distinction is important because the sensing mechanism considered here is electrostatic gating mediated by the EDL. Applying a gate voltage creates an electric double layer (EDL) at the graphene/electrolyte interface, composed of the Helmholtz layer (including adsorbed species such as β_2_M) and the diffuse Gouy–Chapman layer, inducing charge redistribution in graphene and shifting its Fermi level to tune its electronic properties. [49]. This combined effect enhances local electric fields, improves third-harmonic generation efficiency, and enables highly sensitive detection of low biomolecule concentrations, making the system promising for advanced biosensing applications such as protein detection and medical diagnostics [50,51].

Our model depends on the altar in the Fermi energy of the graphene layer generated by a special concentration of the target molecule. To create the concentration-to-optical-response relationship, the β_2_M concentration is first applied to compute the electrostatic potential induced in the electric double layer (EDL) at the graphene/electrolyte interface. This potential behaves as an external gate potential and controls the carrier density of graphene according to

(9) $$n=\frac{C_{\mathrm{EDL}}\phi_{\mathrm{EDL}}}{e},$$

where *C_EDL_* is the EDL capacitance and *e* is the elementary charge. The corresponding Fermi-energy shift is then commuted as

(10) $$E_{F}=\pm \hbar v_{F}\sqrt{\pi \left(n+n^{*}\right)}$$

where *v_F_* is the Fermi velocity and n^*^ is the residual carrier concentration [52,53].

The model which is used for the electric double layer (EDL) is a stern model which is described as below [51,52,53,54]:

(11) $$\frac{1}{C_{\mathrm{EDL}}}=\frac{1}{C_{H}}+\frac{1}{C_{\mathrm{Gc}}}$$

where *C_H_* is its Helmholtz layer and *C_Gc_* is the Gouy–Chapman capacitance, which is computed as below respectively:

(12) $$C_{H}=\frac{\varepsilon_{0}\varepsilon_{H}}{d_{H}}$$

where *ε*_0_ is the permittivity of free space, *ε_H_* the relative permittivity of the electrolyte inside the Helmholtz layer and *d_H_* size of the solvated particle [54].

(13) $$C_{\mathrm{Gc}}=\frac{\varepsilon_{r}\varepsilon_{0}}{\lambda_{D}}\;\cosh(\frac{\mathrm{ze\varphi}}{2K_{B}T})$$

where *λ_D_* is a Debye length given by $\lambda_{D}=({\frac{2ce^{2}}{\varepsilon_{r}\varepsilon_{0}K_{B}T})}^{-\frac{1}{2}}\;$ for a monovalent electrolyte, where *c* is the concentration of the electrolyte, *e* is the electric charge and *K_B_T* is the thermal energy [54].

To reach our aim, we employ an incident wave with an intensity of 0.1 MWcm^−2^ incident, vertically from the top with transverse magnetic (TM) polarization from the first port, while the second port is positioned at the bottom of the structure. Photons with frequency ω are extinguished, and third-order harmonic (TH) radiation with a frequency of 3ω is generated. The thickness of the backside metal, assumed to be gold, is chosen to be much larger than its THz skin depth. As a result, the metallic layer functions as a perfect reflecting mirror, resulting in zero transmission. The optical constants of gold in the terahertz range are described using the Drude model as below [28,55]:

(14) $$\varepsilon_{L,\mathrm{Di}}=\varepsilon_{\infty }-\frac{\omega_{p}^{2}}{\omega^{2}-\mathrm{i\gamma \omega}}$$

where *ω_p_* = 2069 THz is the plasma frequency, *γ* = 17.65 THz is the damping frequency, and *ε_∞_* = 1.53 is the dielectric constant at finite frequency. It should be mentioned that gold shows a third-order nonlinear susceptibility of $\chi_{\mathrm{Au}}^{\left(3\right)}=2.45\times 10^{-19}\;m^{2}V^{-2}$ in the near-infrared region. Meanwhile, its nonlinear principle at low THz frequencies has not been reported in the literature. At these frequencies, gold possesses extremely high conductivity and can be effectively acted as a perfect electric conductor. Consequently, electromagnetic fields do not penetrate the gold, preventing any interaction with its nonlinear properties. In this model, gold acts as a highly reflective metallic boundary, and its intrinsic THG contribution is not considered, with the THG response attributed to graphene. In the next work, ordered and disordered gold gratings will be investigated to explore Anderson localization and its potential for enhancing THG and sensitivity.

Therefore, the contribution of gold to the nonlinear response of the metasurface at low THz frequencies can be considered negligible [56]. This design leverages the unique properties of Graphene, such as its strong nonlinear optical response and tunable bandgap, to enhance third-harmonic generation (THG) and other nonlinear effects in the far infrared and THz frequency ranges. By combining localized plasmon-polariton resonances, and electrical tunability, the proposed metasurface offers significant potential for advanced nonlinear optical applications.

The conversion efficiency (CE) of third-harmonic generation is defined as [34]

(15) $$\mathrm{CE}=\frac{P_{O,\mathrm{TH}}}{P_{i,\mathrm{FF}}}$$

where *P_O_*_,*TH*_ is the output power at the third-order harmonic frequency, and *P_i_*_,*FF*_ is the input power at the fundamental frequency. The output radiation is calculated via

(16) $$P_{O,\mathrm{TH}}=\int (S.n)\mathrm{da}$$

where *S* is the Poynting vector passing through the boundary curve and *n* is the normal vector to the boundary surface. By normalizing the computed power values, which are given in Watts, this assumption enables us to analyze the results on a consistent and physically reasonable scale [57]. Although water exhibits strong absorption in the THz frequency range, THz interactions with aqueous electrolytes can still be analyzed through their dielectric response over short propagation distances. In this work, the dielectric properties of the electrolyte solution are described using the Debye–Falkenhagen theory of ionic atmosphere relaxation, which accounts for the frequency-dependent dielectric behavior of electrolyte solutions. According to this theory, the dielectric constant of the solution increases with the square root of the ion concentration and can be expressed as $\varepsilon_{\mathrm{sol}}=\varepsilon_{H_{2}O}+A\sqrt{c}$, where $\varepsilon_{H_{2}O}$ is the dielectric constant of pure water, *c* is the electrolyte concentration, and *A* is a coefficient dependent on the electrolyte type. The equations used in this study are established formulations adopted from the literature. The novelty of the present study lies in applying graphene-enhanced THG in the THz regime for β2M detection and analyzing the resulting nonlinear optical response to β2M-induced changes in graphene’s electronic properties [58]. A perfectly matched layer (PML) with a thickness of 1.99 × 10^−5^ m was used to decrease artificial reflections at the simulation boundaries. An extremely fine mesh was employed throughout the entire structure, with a maximum element size in the free-space region of 3.331 × 10^−5^/200 m (≈1.67 × 10^−7^ m).

## 3. Results and Discussion

The proposed structure employs a carefully engineered multilayer configuration optimized to enhance third-order harmonic generation (THG). In this design, the interplay between material dispersion, plasmonic response, and field confinement is tuned to maximize nonlinear interaction within the active region. A comprehensive analysis is conducted to evaluate the absorption and reflection spectra and power outflow characteristics. These optical and electromagnetic responses are examined under varying Fermi energy levels, dielectric layer thicknesses, and carrier relaxation times. The performance of the system is studied across a broad frequency range from 1.5 to 3.5 THz, enabling a detailed assessment of how tunable material parameters influence the nonlinear conversion process. This investigation provides valuable insights into optimizing multilayer photonic platforms for high-efficiency THz harmonic generation.

**Figure 3:**
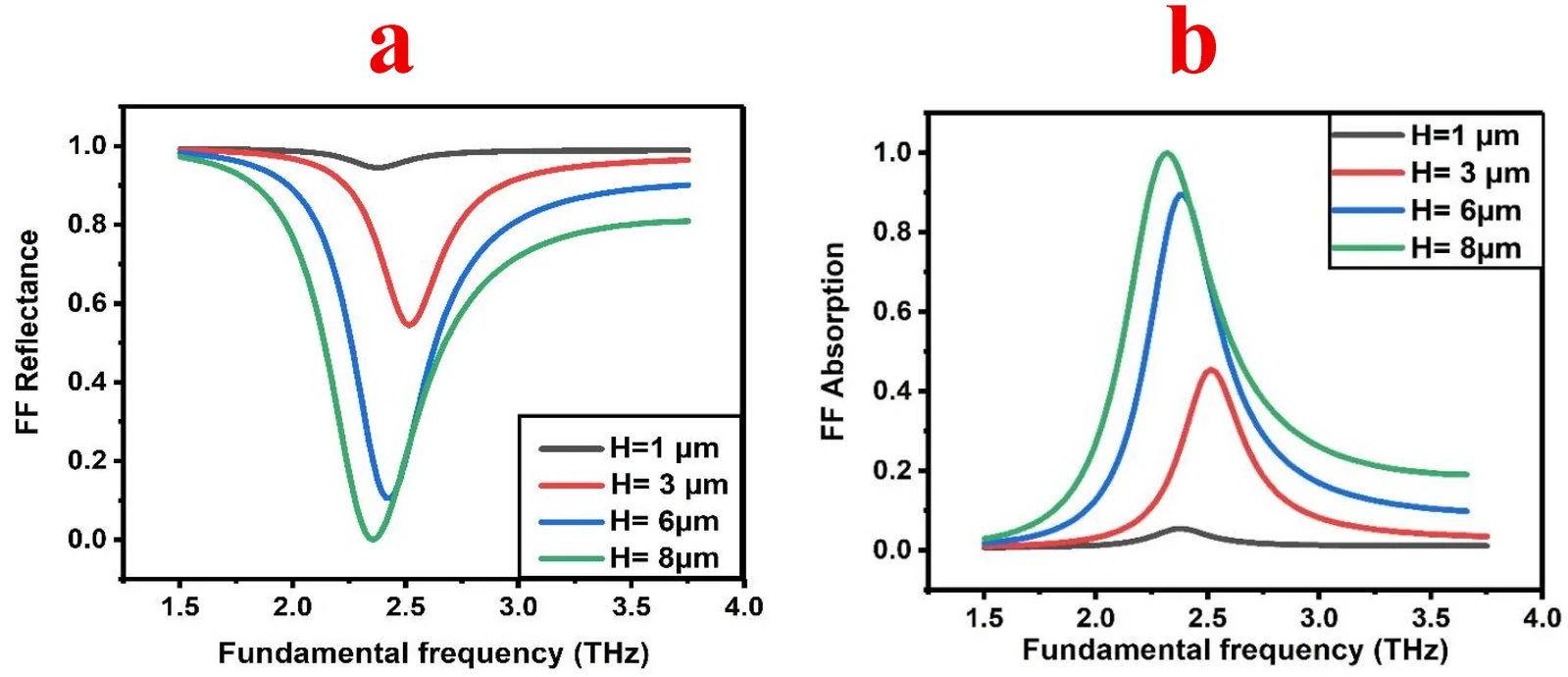

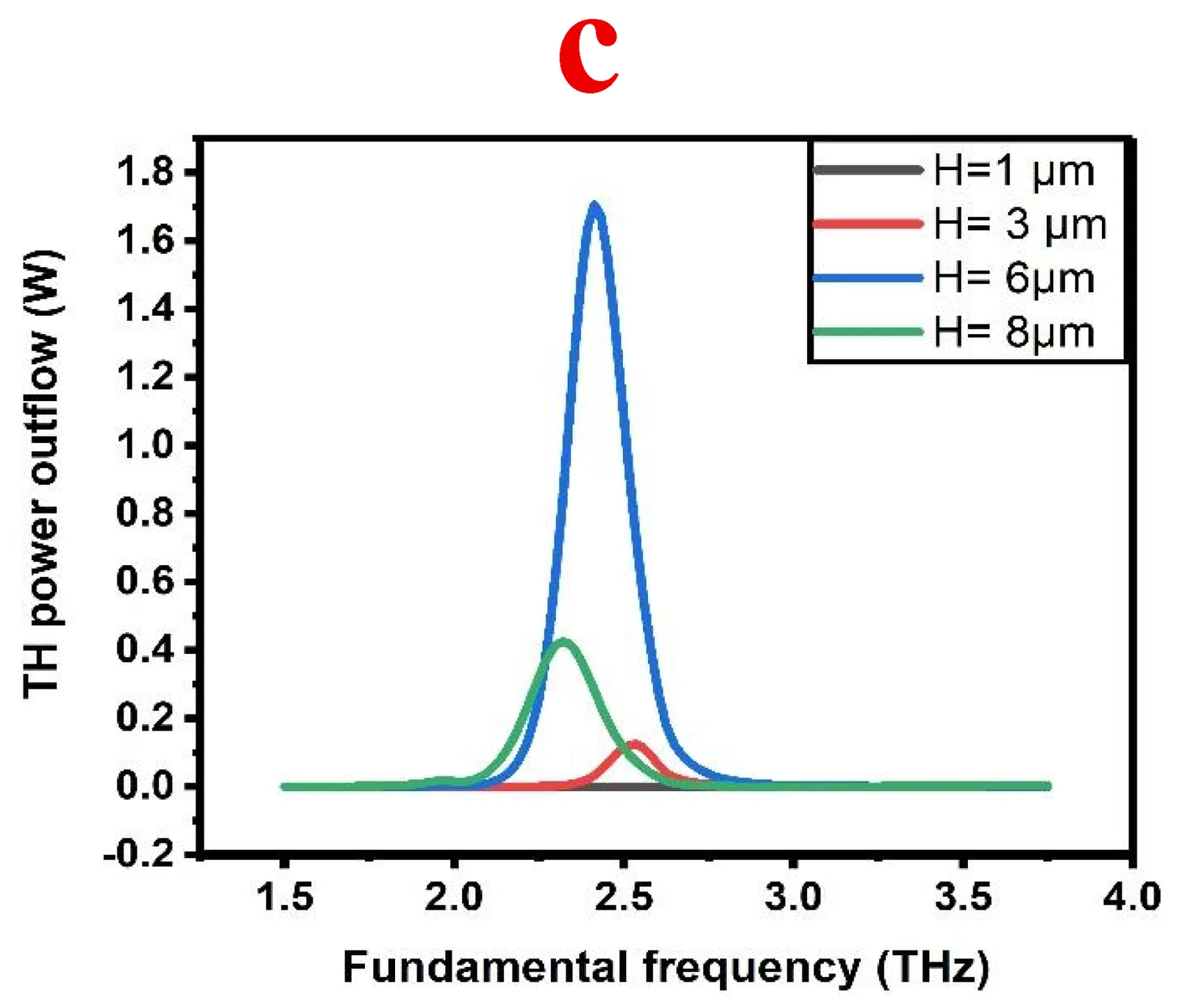
(**a**–**c**) Absorption, reflection, and third-harmonic generation (THG) power outflow spectra for different dielectric thicknesses (**H**) across the frequency range of 1.5–3.5 THz. In all simulations, the graphene relaxation time, Fermi energy of graphene and irritated wave are taken as 0.5 ps, 0.3 eV and 30 $\frac{\mathrm{kW}}{{\mathrm{cm}}^{2}}$ respectively.

To further optimize the nonlinear response of the proposed structure, we investigate the influence of key geometric parameters, with particular emphasis on the thickness of the dielectric layer. For this purpose, the previously analyzed factors—absorption, reflection and power outflow—are re-evaluated for different dielectric thicknesses. Figure 3a–c illustrate the corresponding results, highlighting how variations in dielectric thickness modify field confinement and consequently alter the overall nonlinear performance of the device. These observations provide a deeper understanding of the geometric tuning mechanisms that can be employed to enhance third-order harmonic generation in multilayer THz structures.

As shown in Figure 3, increasing the dielectric height shifts the resonance peak toward lower frequencies. This shift indicates a transition of the resonant frequency to lower energy. The pattern can be attributed to the decreased vertical confinement of the plasmonic modes and the redistribution of the local electromagnetic field, which effectively increases the optical mode volume and reduces the resonance energy.

**Figure 4:**
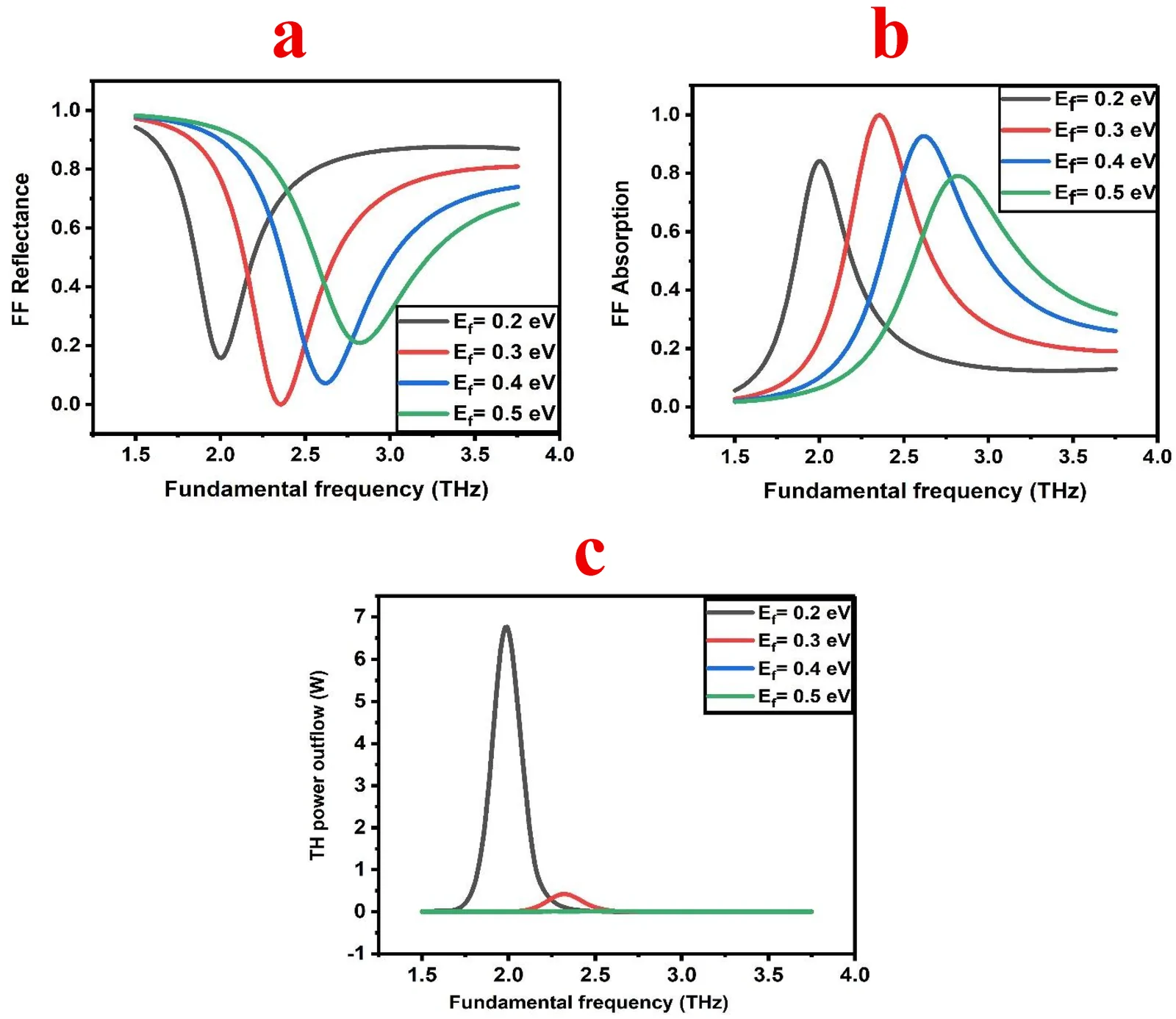
(**a**–**c**) Absorption, reflection, and third-harmonic generation (THG) power outflow spectra for different Fermi energy of graphene (*E_f_*) across the frequency range of 1.5–3.5 THz. In all simulations, the graphene relaxation time, thickness of dielectric and irritated wave are taken as 0.5 ps, 8 um and 30 $\frac{\mathrm{kW}}{{\mathrm{cm}}^{2}}$ respectively.

The absorption, reflectance, and TH power outflow spectra of the structure are analyzed for various Fermi energies—Ef = 0.2, 0.3, 0.4, 0.46, and 0.5 eV—across the same frequency range, with the corresponding results presented in Figure 4a–c. It is evident that each Fermi energy level produces a distinct resonance associated with the excitation of surface plasmon polaritons. This pattern demonstrates that graphene absorbs light at specific frequencies, thereby reducing the transmission of the fundamental field. As Fermi energy increases, the electron population in higher energy states also increases, resulting in a shift of both the resonance frequency and the transmission minimum toward higher values. In these simulations, Fermi energies of 0.2 eV, 0.3 eV, 0.4 eV, and 0.5 eV were used, while the relaxation time was fixed at 0.5 ps and the dielectric thickness at 8 µm. Figure 4a–c illustrate the absorption, reflectance, and TH power outflow of graphene at different Fermi energies, respectively.

**Figure 5:**
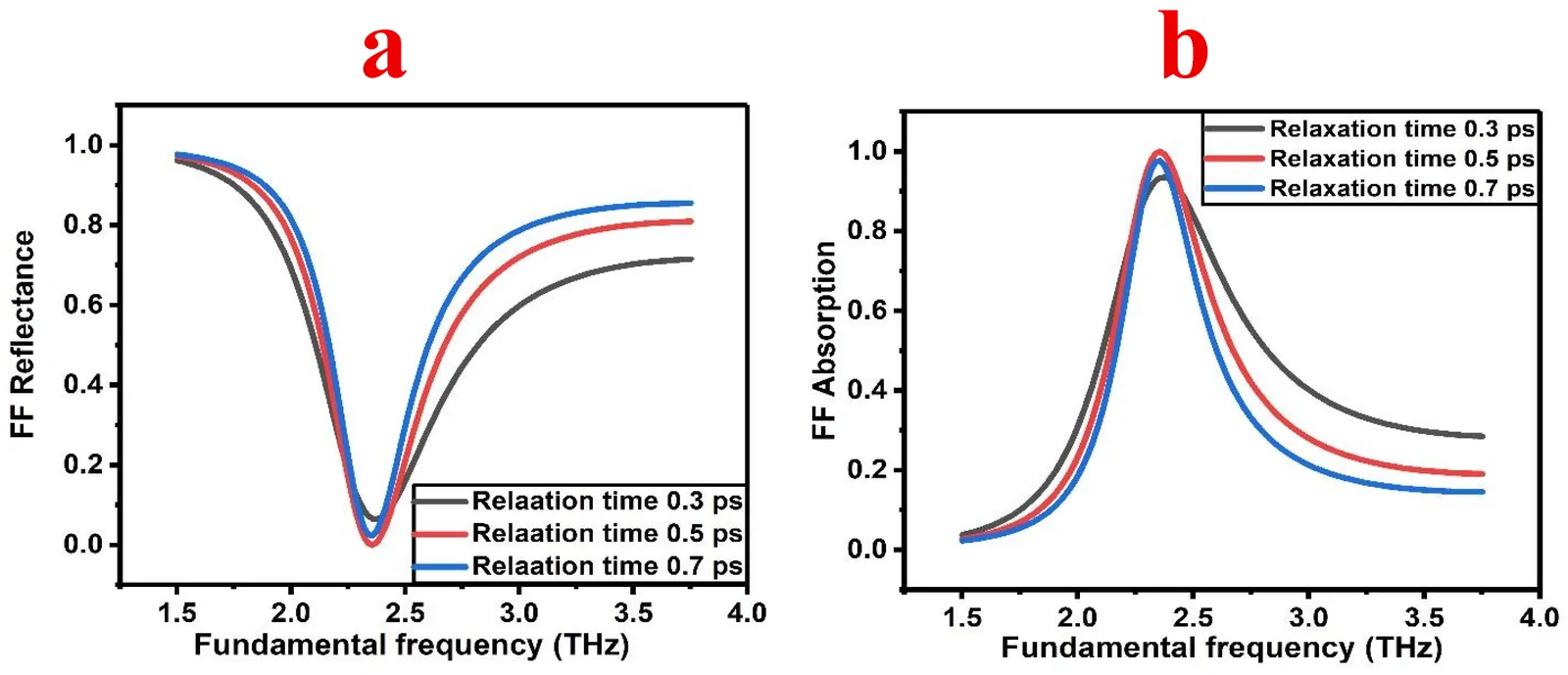

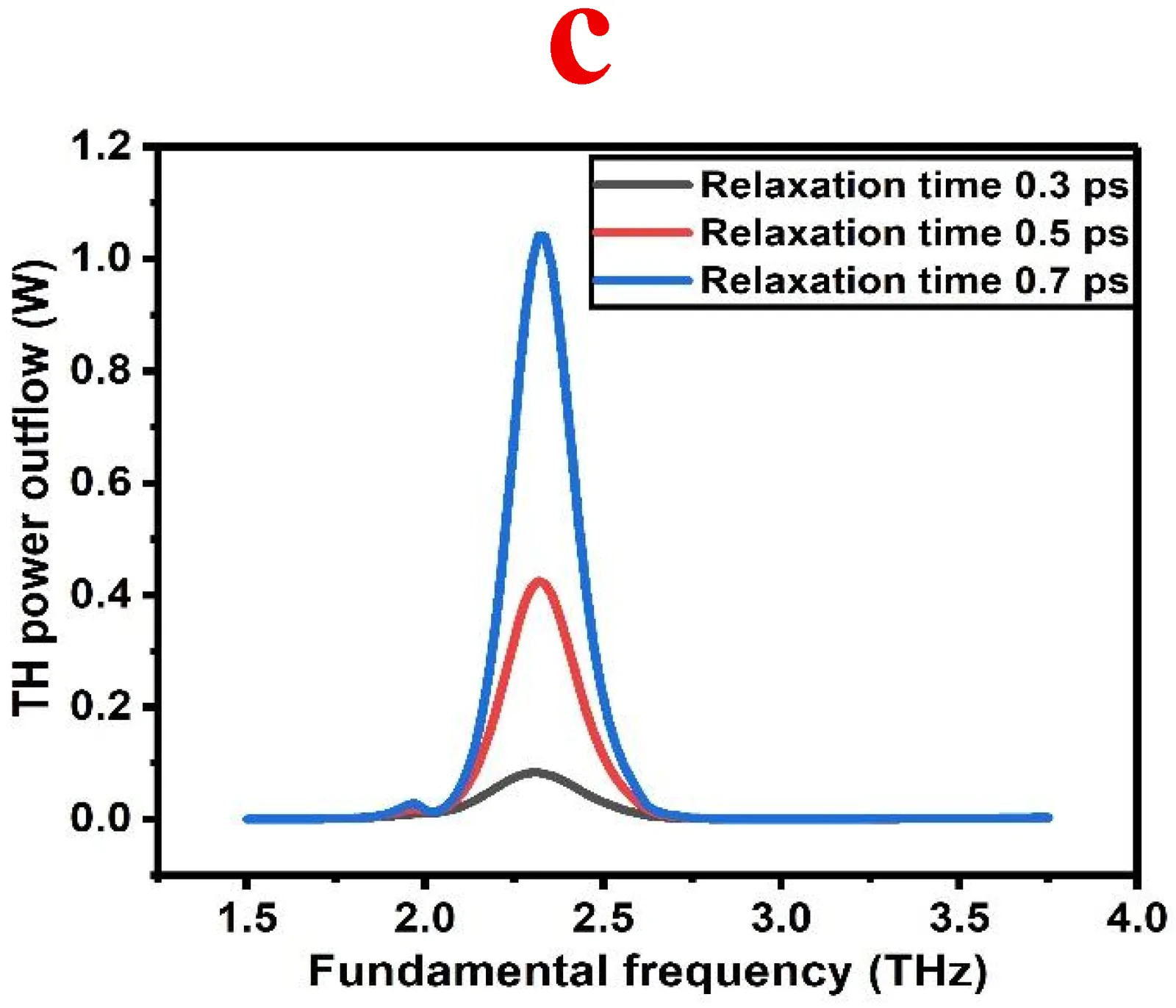
(**a–c**) Absorption, reflection, and third-harmonic generation (THG) power outflow spectra for different relaxation time (*τ*) of graphene microribbon across the frequency range of 1.5–3.5 THz. In all simulations, the thickness of dielectric, Fermi energy of graphene and irritated wave are taken as 8 um, 0.3 eV and 30 $\frac{\mathrm{kW}}{{\mathrm{cm}}^{2}}$ respectively.

In the next step of our optimization, we investigate the optical performance of the sensor under different carrier mobilities. To achieve this, we use the relationship between relaxation time and the carrier mobility of graphene, as described by Equation (3). This equation indicates a direct correlation between these parameters. Accordingly, in this computation, we apply three different relaxation times: 0.3 ps, 0.5 ps, and 0.7 ps. Throughout this analysis, the Fermi energy is fixed at 0.3 eV, and the dielectric thickness is maintained at 8 µm. Figure 5a–c illustrate the absorption, reflectance, and TH power outflow of graphene at different relaxation times, respectively.

**Figure 6:**
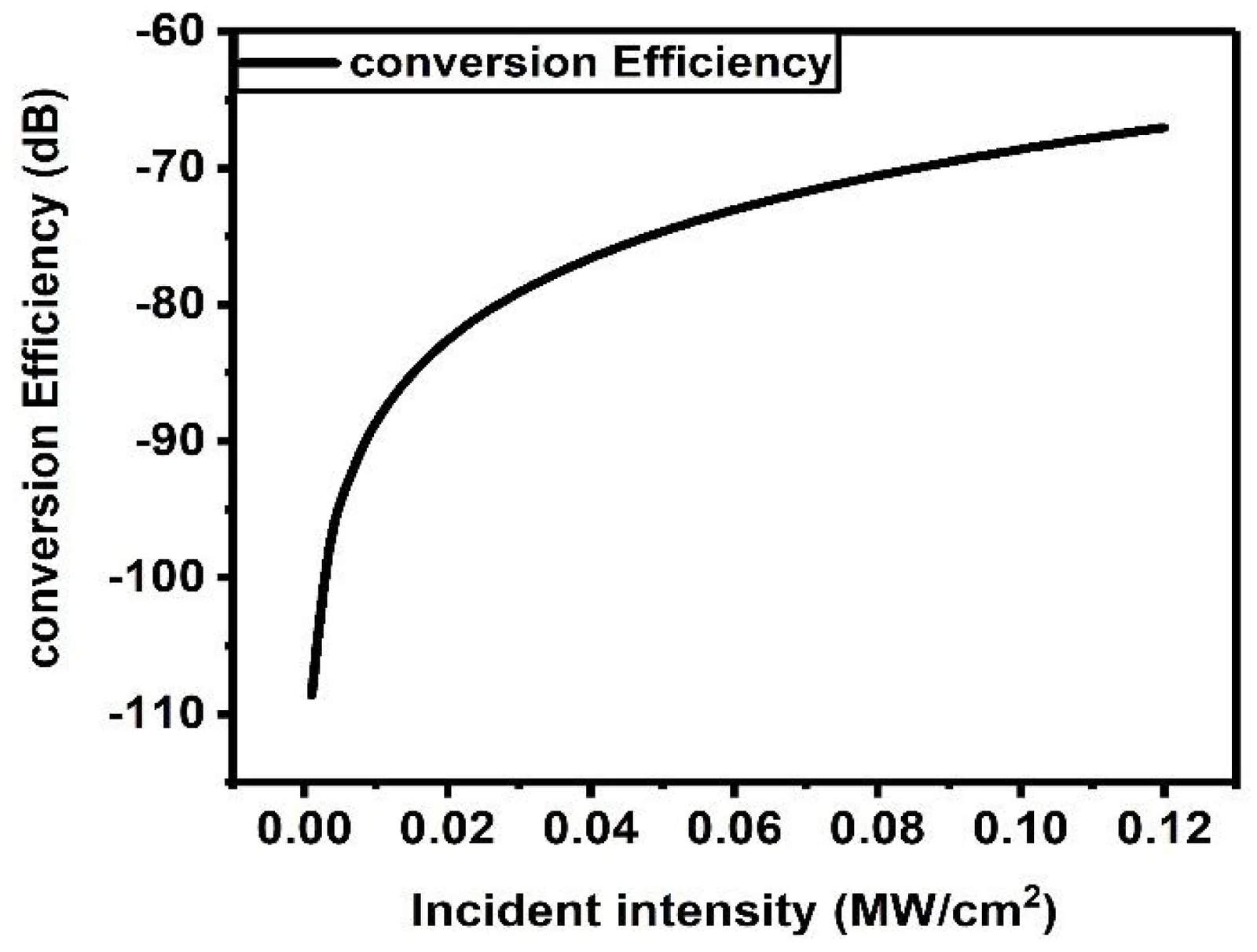
Computed THG conversion efficiency as functions of the incident FF wave intensity at fundamental frequency of 3 THz. In all simulations, the graphene relaxation time, Fermi energy of graphene and thickness of dielectric are taken as 0.5 ps, 0.3 eV and 8 um respectively.

To study the impact of the input field intensity on the generated field power and conversion efficiency, the input intensity was varied from 0.001 to 0.12 MW·cm^−2^ at a fixed frequency of 3 THz, as shown in Figure 6. Higher input intensities increase the number of photons in the structure, thereby enhancing the probability of three photons at frequency **ω** interacting to generate a photon at frequency 3ω. Interestingly, the THG conversion efficiency can reach relatively high values (−65 dB) even at comparatively low fundamental-field input intensities when exciting the proposed nonlinear graphene metasurface. Figure 6 illustrates this variation. In these simulations, the parameters used were a Fermi energy of 0.3 eV, a relaxation time of 0.5 ps, and a dielectric thickness of 8 µm.

**Figure 7:**
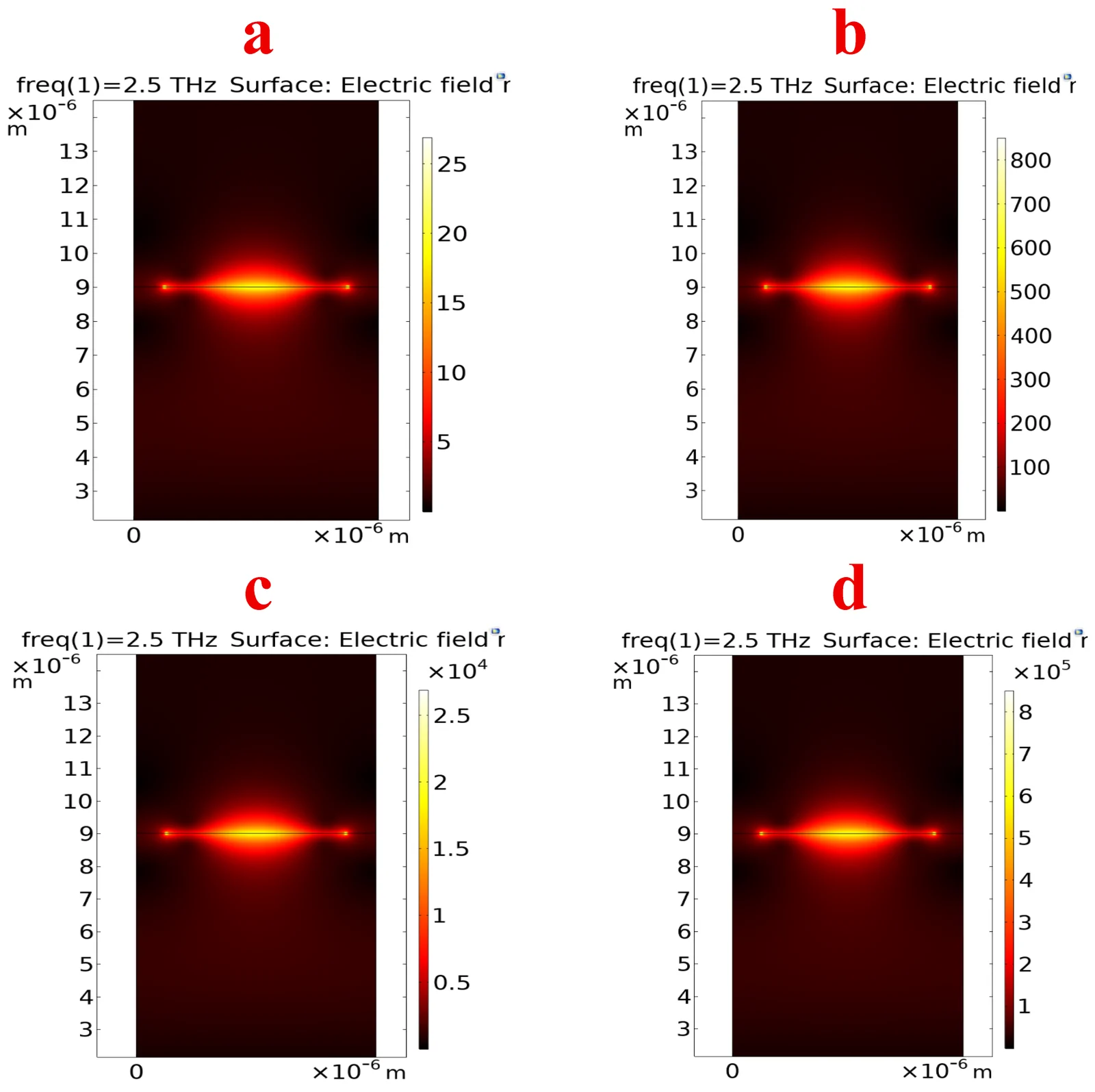
Normalized spatial distribution of the electric field (V/m) for the third-harmonic generation wave at the plasmon resonance frequency of 1.5 THz for different intensity of irritated (*I*_0_) wave in graphene microribbon: (**a**) $I_{0}=0.1\frac{\mathrm{kW}}{{\mathrm{cm}}^{2}}$, (**b**) $I_{0}=1\frac{\mathrm{kW}}{{\mathrm{cm}}^{2}}$, (**c**)$\;I_{0}=10\frac{\mathrm{kW}}{{\mathrm{cm}}^{2}}\;$ and (**d**) $I_{0}=100\frac{\mathrm{kW}}{{\mathrm{cm}}^{2}}$. In all simulations, the Fermi energy of graphene, dielectric high and relaxation time are taken as 0.3 eV, 8 μm and 0.5 ps respectively.

Figure 7a–d illustrate the electric field enhancement under different incident light intensities, demonstrating the relationship between light intensity and field enhancement. As the incident light intensity increases, the electric field enhancement in the graphene-based THG structure also increases. This result indicates that higher excitation intensities lead to stronger electric field localization, thereby enhancing the third-harmonic generation (THG) response of the graphene structure.

In this section, we examine how the proximity of a protein molecule alters the electrostatic environment adjacent to a graphene surface. Building on the theoretical framework introduced earlier, the evaluation of the electric field and potential inside the electric double layer (EDL) begins with the Poisson equation. After establishing the computational domain and boundary conditions, a set of numerical simulations was carried out to extract and compare the relevant electrostatic profiles. These graphical outputs enabled a detailed assessment of the modifications introduced when quantum capacitance is incorporated into the model, particularly regarding the spatial variation of potential and field strength.

Within the compact layer, the electrostatic pattern was determined by solving the Poisson equation, (*εε*_0_∇*φ*) = 0. In contrast, transport in the diffuse region was described using a coupled Nernst–Planck–Poisson formulation to provide a more realistic depiction of ion and protein motion [59,60,61,62,63,64,65]:

(17) $$\frac{{\partial c}_{i}}{\partial t}=\nabla (D_{i}\nabla c_{i}+z_{i}FD_{i}c_{i}\nabla \frac{\varphi }{\mathrm{RT}})$$

together with

∇(*εε*_0_∇*φ*) = *ρ* (18)

Here, *D_i_*, *z_i_*, and *c_i_* represent the diffusivity, charge valence, and concentration of species *i*; *t* is time; *F* is the Faraday constant; *R* is the universal gas constant; *T* is the temperature; *φ* is the electrostatic potential; *ε* and *ε*_0_ are the medium and vacuum permittivities; and *ρ* denotes the net charge density.

**Figure 8:**
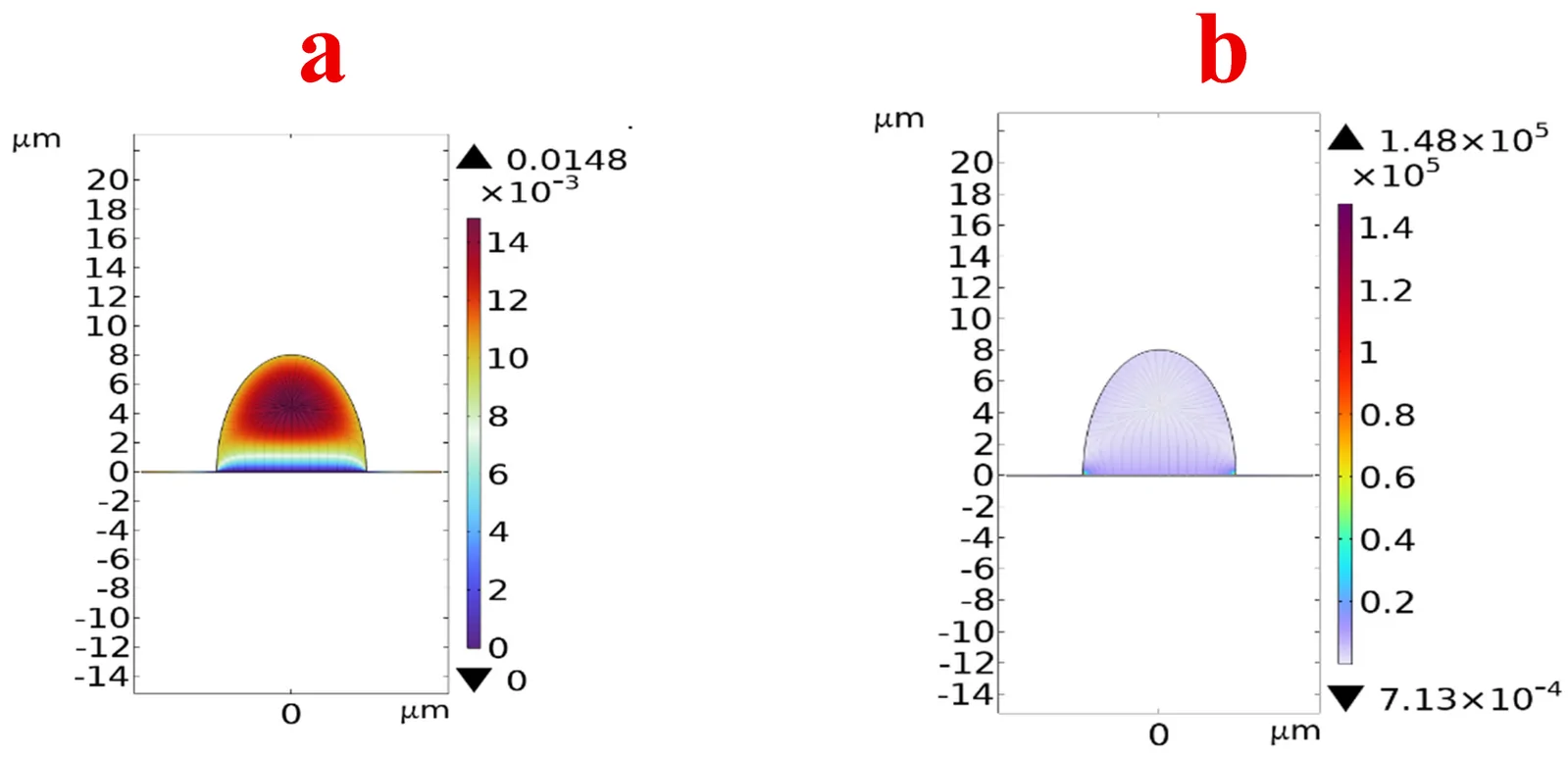
Panels (**a**,**b**) show the electric potential (**a**) and electric field (**b**) in the electrolyte, including the region near the electric double layer (EDL). The simulation uses the Stern model of EDL integrated with quantum capacitance of Graphene.

Figure 8a,b present the resulting distributions, highlighting the corresponding potential and electric-field patterns formed within the EDL.

**Figure 9:**
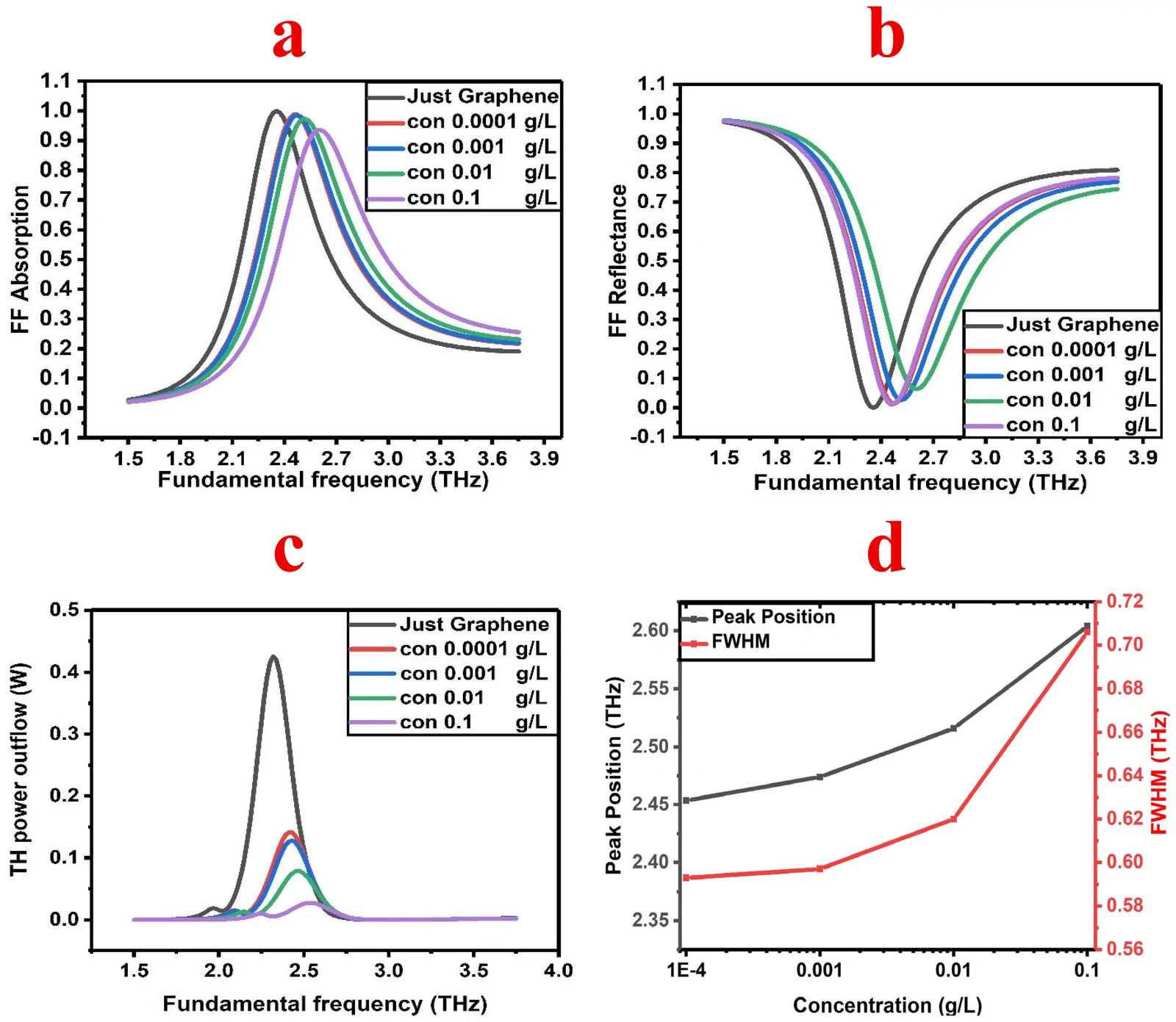
Absorption (**a**), (**b**) reflection, and (**c**) third-harmonic generation (THG) power outflow spectra and (**d**) FWHM for different protein concentration across the frequency range of 1.5–3.5. In all simulations, the graphene relaxation time, Fermi energy of graphene, irritated wave intensity and thickness of dielectric are taken as 0.5 ps, 0.3 eV, 30 $\frac{\mathrm{kW}}{{\mathrm{cm}}^{2}}$ and 8 um respectively.

In this step, we compute the absorption, reflectance, and third-harmonic power outflow for different concentrations of β2-microglobulin. As discussed in the previous chapter, the presence of this protein in its wild-type form carries a net charge of −2. This negative charge induces electron doping in graphene, leading to an increase in its Fermi energy. Consequently, the optical response of graphene is modified, resulting in a noticeable shift of the absorption and reflectance peaks toward higher frequencies. Figure 9 illustrates this pattern clearly, where increasing the concentration of β2-microglobulin produces a systematic blue shift in the spectral features. This shift arises from the strong coupling between the charged biomolecules and the graphene surface, which alters the carrier density and thus the nonlinear optical properties of the system. Moreover, the enhancement in Fermi energy significantly influences the third-harmonic generation process, leading to changes in the magnitude of the third-harmonic power outflow. These results highlight the sensitivity of graphene’s linear and nonlinear optical responses to biomolecular adsorption, underscoring its potential application as a highly sensitive biosensing platform. In this study, (0.1 mg/L) is considered the lowest simulated concentration of β_2_M, rather than a formal limit of detection. This concentration was selected to evaluate the response of the proposed sensing platform at a low, clinically relevant concentration. A true experimental detection limit would require experimental measurements and statistical analysis.

**Figure 10:**
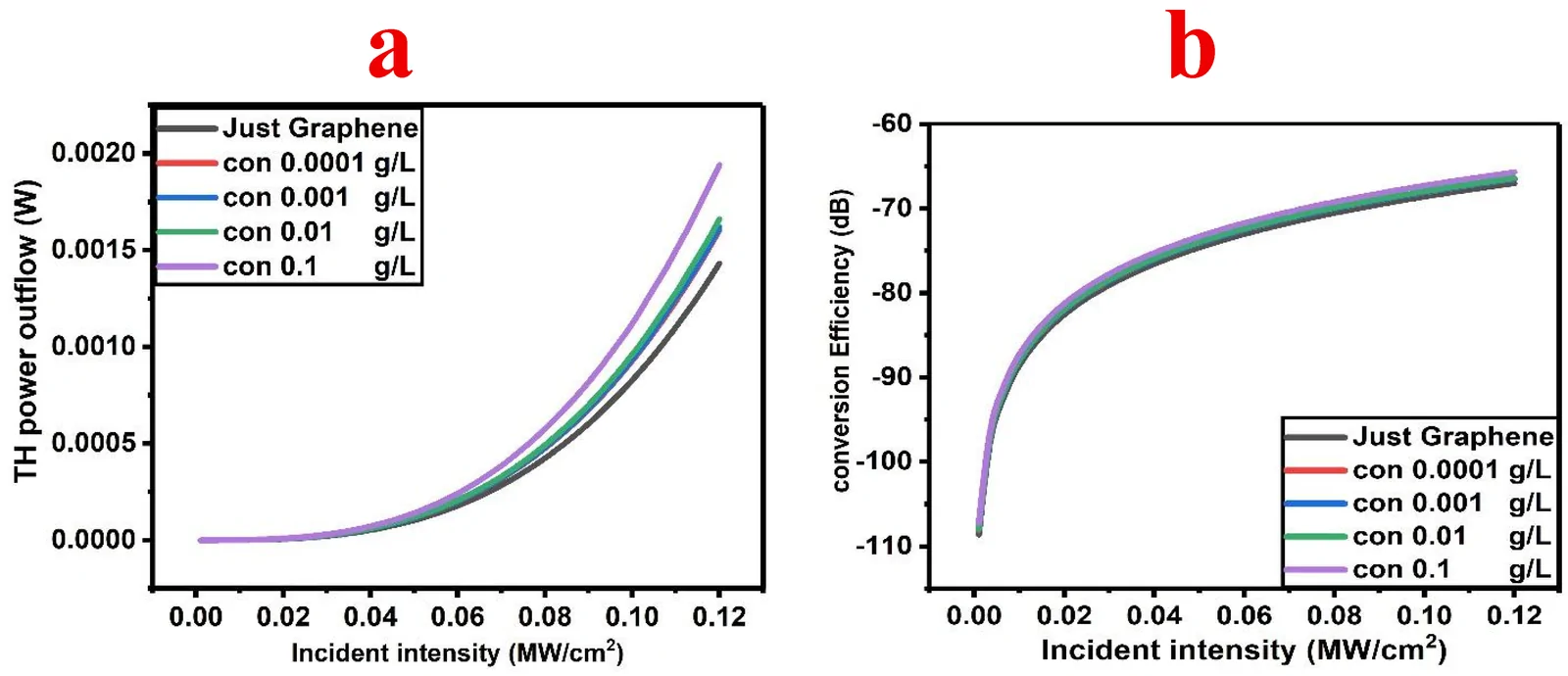
Effect of incident intensity on (**a**) conversion efficiency and (**b**) third-harmonic generation power outflow as functions of the incident wave intensity at fundamental frequency of 3 THz with different concentrations of protein. In all simulations, the graphene relaxation time, Fermi energy of graphene and thickness of dielectric are taken as 0.5 ps, 0.3 eV and 8 um respectively.

In the final step of our computation, we investigate the conversion efficiency and third-harmonic power outflow as functions of the incident optical intensity. For this purpose, the input intensity is varied from 0.001 MW/cm^2^ to 0.12 MW/cm^2^ for different concentrations of the biomolecule. As shown in Figure 10, increasing the incident intensity leads to pronounced variations in both the conversion efficiency and the third-harmonic output.

This pattern is attributed to the nonlinear optical response of graphene, where higher input intensities enhance the nonlinear polarization responsible for third-harmonic generation. These results demonstrate that both incident intensity and molecular concentration are key factors in optimizing third-harmonic generation, reinforcing the potential of this system for tunable nonlinear optical and biosensing applications. Figure 10 shows the conversion efficiency and TH power outflow in different incident intensities.

To address in a quantitative fashion the performance of our sensor—namely, sensitivity, accuracy and reliability—we use the following parameters:

(19) $$S=\frac{\Delta f}{\Delta C}$$

(20) $$\mathrm{FOM}=\frac{S}{\mathrm{FWHM}}$$

(21) $$Q=\frac{f_{r}}{\mathrm{FWHM}}$$

(22) $$\mathrm{SNR}=\frac{\Delta f}{\mathrm{FWHM}}$$

(23) $$\mathrm{DA}=\frac{1}{\mathrm{FWHM}}$$

Here, *f* denotes the frequency, *c* represents the protein concentration, *f_r_* is the peak position of absorption, *S* indicates the sensitivity, *FWHM* refers to the full width at half maximum, *Q* is the quality factor, *FOM* stands for the figure of merit, *SNR* denotes the signal-to-noise ratio, and *DA* represents detection accuracy [33].

The quantity Δ*f*/FWHM is used throughout this study and in Table 1 as the normalized resonance shift, representing the resonance displacement relative to its linewidth; however, it does not account for noise and therefore cannot be interpreted as a physical signal-to-noise ratio. The calculated sensitivity (971 THz·L/g) and figure of merit (FOM = 1706 L/g) are based on deterministic simulations and indicate a strong theoretical response but do not independently demonstrate experimentally validated sensing performance or improved detection capability over linear sensing. Accordingly, the lowest considered concentration of 0.0001 g/L (0.1 mg/L) is reported as the lowest numerically investigated concentration rather than an experimentally established detection limit. Practical detection may be limited by detector and readout noise, excitation-intensity and frequency fluctuations, temperature variations, and graphene carrier-density fluctuations associated with instability at the graphene–electrolyte interface. Since the simulations do not account for stochastic measurement noise or experimental variability, they cannot establish the actual detection limit or identify the dominant noise source. Therefore, experimental noise characterization, repeated measurements of blank and low-concentration samples, and statistical validation are necessary to determine the practical detection limit and verify the sensor’s real-world performance.

By employing third-harmonic generation (THG) in our sensor design, we achieved a sensitivity of 117.5 THz·L/g and a figure of merit (FOM) of 198 L/g at a β2-microglobulin concentration of 0.001 g/L. In contrast, under the same operating conditions, the sensor operating in the linear regime exhibited a sensitivity of 84.74 THz·L/g and an FOM of 51 L/g [33]. These results demonstrate that the THG-based approach significantly enhances both the sensitivity and overall sensing performance compared to the conventional linear regime. We acknowledge that a direct quantitative comparison between linear and THG sensing under identical structural and analyte conditions was not performed. Meanwhile, the THG results are shown as demonstrating its potential, while a systematic comparison under same conditions is planned as future work.

Consequently, the proposed nonlinear sensing strategy offers improved detection capabilities for β2-microglobulin, making it a promising platform for highly sensitive biomarker detection and biomedical diagnostic applications. The results presented in Figure 3, Figure 4, Figure 5, Figure 6, Figure 7, Figure 8, Figure 9 and Figure 10 are based on numerical simulations and have not yet been experimentally validated. Therefore, the predicted sensing performance should be considered theoretical and require experimental verification. Experimental validation and characterization of the proposed sensor will be pursued in future work.

## 4. Conclusions

In conclusion, this work presents a nonlinear ultrathin graphene metasurface for efficient third-harmonic generation (THG) at terahertz (THz) frequencies under relatively low excitation intensities. The proposed structure combines graphene plasmonic resonances with Fabry–Pérot cavity resonances in the dielectric layer, producing strong electromagnetic field confinement and significantly enhancing the nonlinear response. Owing to graphene’s strong third-order nonlinear conductivity in the THz regime, the metasurface achieves a THG conversion efficiency of approximately −65 dB at an incident intensity of ~0.1 MW·cm^−2^, demonstrating its potential for low-power nonlinear THz applications.

The proposed metasurface also provides high tunability and reconfigurability through control of the graphene Fermi energy, dielectric thickness, and carrier relaxation time. These parameters enable effective control of the resonance frequency, nonlinear conductivity, and optical losses, allowing the THG response to be engineered for different operating frequencies. Such flexibility makes the proposed platform suitable for compact and adaptive THz nonlinear optical devices.

Furthermore, the sensing performance was investigated using β2-microglobulin (β2M) as a target biomolecule. Increasing β2M concentration produces a measurable shift of the resonance toward higher frequencies due to the negative charge of the protein and its interaction with graphene, which modifies the effective Fermi energy and plasmonic response. This concentration-dependent behavior demonstrates the strong sensitivity of the nonlinear metasurface to biochemical changes.

Overall, the proposed graphene-based THG metasurface offers a compact, efficient, tunable, and highly sensitive platform for THz frequency conversion and biosensing, with promising applications in integrated THz photonic systems, biomolecular detection, and biomedical diagnostics.

## Figures and Tables

**Table 1:** Key parameters influencing the performance of the sensor in different concentrations of β_2_-macroglobulin in nonlinear optics.

Sensor Parameters’ Performance	β2-M C = 0.0001 g/L	β2-M C = 0.001 g/L	β2-M C = 0.01 g/L	β2-M C = 0.1 g/L
S (THz·L/g)	971	117.500	15.932	2.473
FOM (L/g)	1706	198	25	3.58
Q	4.31	4.17	3.99	3.77
SNR	0.170	0.198	0.253	0.358
DA (1/THz)	1.75	1.68	1.58	1.44

## Data Availability

The original contributions presented in this study are included in the article/Supplementary Material. Further inquiries can be directed to the corresponding author.

## Supplementary Materials

The following supporting information can be downloaded at: https://www.mdpi.com/article/10.3390/s26216779/s1.
