# Supplementary material for: Nonlinear Optical Detection of β2-Microglobulin Exploiting Graphene-Enhanced Terahertz Third-Harmonic Generation
Source: Sensors (Basel). 2026 Oct 26;26(21):6779. doi: 10.3390/s26216779 (PMC13765795; doi:10.3390/s26216779)
Supplement: Supplementary file 1 [file sensors-26-06779-s001.zip › sensors-4590814-supplementary.pdf]

Nonlinear Optical Detection of β2-Microglobulin exploiting Graphene-Enhanced terahertz Third-Harmonic Generation

Ghassem Baridi [1], Arslan Liaquat [2], Federico Rapuzzi [2], Herath Mudiyanselage Kasun Gayanga Anuradha Herath [2], Maria Celeste Maschio [2,3], El Hadj Abidi [4], Vito Clericò [4], Yahya Moubarak Meziani [5], Mario Amado [5], Enrique Diez [5], Stefano Corni [3], Giorgia Brancolini [3], Luigi Rovati [1] and Francesco Rossella [2]

[1]Department of Engineering "Enzo Ferrari", University of Modena and Reggio Emilia, Via P. Vivarelli, 10, 41125 Modena, Italy

[2]Department of Physics, Computer Science and Mathematics, University of Modena e Reggio Emilia, Via Campi 213/a, I-41125 Modena, Italy

[3] Istituto Nanoscienze - CNR, S3, Via G. Campi 213/A, Modena, Italy

[4]Department of Applied Physics, University of Salamanca, 37008 Salamanca, Spain

[5] Nanotechnology Group, USAL–Nanolab, Departamento de Física Fundamental, University of Salamanca, Plaza de la Merced, Edificio Trilingüe, 37008, Salamanca, Spain

In this study, a label-free graphene-based sensing mechanism for β2-microglobulin (β2M) is investigated based on the electrostatic interaction between the protein and the graphene surface. β2M is modeled as a charged macromolecule, and its concentration-dependent electrostatic potential near the graphene surface is calculated using the Poisson equation, considering parameters specific to β2M, including its molecular size, effective charge, and concentration. The resulting electric field modifies the carrier concentration and Fermi level of graphene, leading to a corresponding change in its optical response. Thus, the present work focuses on establishing the fundamental transduction mechanism from β2M concentration to electrostatic potential, graphene Fermi-level modulation, and optical response, rather than demonstrating molecular selectivity in a complex biological matrix. Since bare graphene can also interact with other charged proteins and ions, selective recognition of β2M is not claimed in the present study. Future work will focus on functionalizing the graphene surface with a β2M-specific recognition element, followed by systematic interference studies with relevant proteins and other species present in biological samples, to develop a highly selective β2M sensing platform.

To establish the concentration-to-optical-response relationship, the β_2M concentration is first used to calculate the electrostatic potential generated in the electric double layer (EDL) at the graphene/electrolyte interface. This potential acts as an external gate potential and modifies the carrier density of graphene according to

$$n = \frac{C_{\mathrm{EDL}} \phi_{\mathrm{EDL}}}{e},$$

where $C_{\mathrm{EDL}}$is the EDL capacitance and $e$is the elementary charge. The corresponding Fermi-energy shift is then calculated as

$$E_F = \pm \hbar v_F \sqrt{\pi (n + n^*)},$$

where $v_F$is the Fermi velocity and $n^*$is the residual carrier concentration. Thus, the concentration-dependent optical response follows the sequence [63]:

$$C_{\beta 2M} \rightarrow \phi_{\mathrm{EDL}} \rightarrow n \rightarrow E_F \rightarrow \text{optical response.}$$

The calculated EDL potentials, induced carrier densities, Fermi energies, and corresponding optical shifts for the different β2M concentrations are summarized in **Table S1**. In the simulations, graphene initially has a Fermi energy of 0.3 eV, and the concentration-dependent EDL potential is subsequently included to determine the modified Fermi energy. This approach establishes the physical connection between β2M concentration and the observed optical resonance shift.

**β2-M concentration**	**Potential near to Graphene surface (mV)**	**Amount of Fermi energy of graphene (eV)**
.0001 [g/L]	**188.77**	**.346**
**.001 [g/L]**	**181.56**	**.35**
**.01 [g/L]**	**167**	**.369**
**.1 [g/L]**	**136**	**.395**

Numerical Model and Simulation Details

To ensure the reproducibility of the numerical results presented in this work, the complete details of the electromagnetic model, boundary conditions, excitation conditions, nonlinear model, mesh, and power calculations are provided here.

Simulation geometry and boundary conditions

The proposed metasurface is modeled using a **two-dimensional frequency-domain electromagnetic model**. Owing to the periodic nature of the structure, only one unit cell containing a single graphene ribbon is considered in the computational domain. The graphene ribbon has a width of

$$d = 0.2\ \mu\text{m}.$$

Since the model is two-dimensional, the graphene ribbon is assumed to have a length of 1 m in the y-direction. The graphene layers are modeled as infinitesimally thin conductive sheets and are therefore treated as **surface currents** rather than volumetric materials. Their physical sub-nanometer thickness is neglected in the numerical model.

Floquet periodic boundary conditions are applied to the lateral boundaries perpendicular to the x-axis. These boundary conditions reproduce the response of the periodically repeated metasurface from a single unit cell.

The complete geometrical parameters of the unit cell, including the periodicity, dielectric-layer thicknesses, graphene-layer separation, and total computational-domain dimensions, are defined according to the geometry of the proposed structure.

Excitation and port configuration

The structure is illuminated by a normally incident fundamental-frequency electromagnetic wave with an input intensity of

$$I_{\text{in}} = 30\ \text{kW/m}^2.$$

The incident wave is TM-polarized and is introduced through the upper port. The second port is positioned at the bottom of the computational domain. The upper and lower ports are used to determine the reflected and transmitted electromagnetic powers, respectively.

The incident port excitation is normalized to the input intensity of $30\ \mathrm{kW/m^2}$. The reflected and transmitted powers are subsequently normalized with respect to the incident power at the excitation port.

Graphene layers and nonlinear contribution

Three graphene layers are considered in the proposed structure. Since graphene is represented as a surface current, its finite physical thickness is not explicitly included in the two-dimensional geometry.

The factor $N = 3$appearing in Eq. (8) represents the number of graphene layers in the proposed structure. It accounts for the combined contribution of the three graphene sheets under the assumption that the graphene layers experience the same electromagnetic conditions used in the nonlinear calculation.

The nonlinear Kerr coefficient used for the graphene-related nonlinear contribution was obtained from a hybrid waveguide consisting of silicon and monolayer graphene. The nonlinear coefficient calculated from the four-wave-mixing spectra is

$$n_2 = 4.8 \times 10^{-17}\ \mathrm{m^2/W}$$

at a wavelength of approximately $1.56\ \mu$m. This value is used to characterize the nonlinear response associated with the graphene-based hybrid structure.

The nonlinear refractive index is described by

$$n = n_0 + n_2 I,$$

where $n_0$is the linear refractive index, $n_2$is the Kerr nonlinear coefficient, and $I$is the local optical intensity.
It should be noted that the dielectric spacer itself is treated as a linear material in the present

simulations. Therefore, the nonlinear contribution considered in the calculations originates from the graphene-based nonlinear response. Accordingly, the terminology “Kerr nonlinear dielectric spacer” has been removed from the manuscript to avoid ambiguity and to ensure consistency between the physical model and the numerical implementation.

Mesh

A highly refined nonuniform mesh is employed throughout the computational domain, with additional local refinement near the graphene surfaces and graphene/dielectric interfaces where strong electromagnetic-field variations may occur.

The maximum element size in the free-space region is

$$h_{\text{free}} = 3.88\ \mu\text{m} = 3880\ \text{nm}.$$

The mesh is further refined around the graphene edges and within the dielectric regions. The corresponding local mesh sizes are selected to accurately resolve the electromagnetic fields near the graphene/dielectric interfaces.

A mesh-convergence analysis is performed by progressively refining the mesh and comparing the resulting electromagnetic spectra. The final mesh is selected when further mesh refinement produces no appreciable change in the calculated resonance position and transmission/reflection response.

Frequency-domain solution and nonlinear calculation

The linear and nonlinear electromagnetic responses of the metasurface are calculated using the frequency-domain electromagnetic solver. The incident fundamental-frequency field is applied through the upper port with an intensity of $30\ \text{kW/m}^2$. The nonlinear electromagnetic response is obtained by incorporating the nonlinear constitutive relation into the frequency-domain electromagnetic calculation.

The numerical solver tolerances and nonlinear convergence criteria used in the calculations are specified according to the solver settings employed in the simulations.

Calculation of reflected and transmitted powers

The reflected and transmitted powers are calculated from the time-averaged Poynting vector. The time-averaged electromagnetic power passing through a surface S is calculated according to

$$P = \int_S \frac{1}{2} \mathrm{Re}(\boldsymbol{E} \times \boldsymbol{H}^*) \cdot \boldsymbol{n} \, dS,$$

where $\boldsymbol{E}$ and $\boldsymbol{H}$ are the complex electric and magnetic fields, respectively, $\boldsymbol{H}^*$ denotes the complex conjugate of the magnetic field, and $\boldsymbol{n}$ is the outward normal vector to the integration surface.

The reflected power is obtained from the upper port, while the transmitted power is obtained from the lower port. The corresponding reflection and transmission coefficients are calculated by normalizing these powers with respect to the incident power.

Reproducibility and convergence

To verify the numerical reliability of the calculated results, mesh refinement and computational-domain convergence tests are performed. The mesh is progressively refined, particularly around the graphene layers and dielectric interfaces, and the resulting spectra are compared. Similarly, the computational domain is sufficiently extended from the metasurface to ensure that further changes in the domain size do not produce an appreciable modification of the calculated response.

The numerical parameters described above, together with the complete geometrical dimensions, dielectric material properties, frequency-dependent dispersion data, graphene-layer separation, local mesh sizes, solver tolerances, and convergence results, are provided to facilitate independent reproduction of the reported simulations.

A new subsection has been added to the Supporting Information to provide the boundary conditions and numerical-model details required for reproducibility. The treatment of the periodic boundaries and the corresponding conditions used for the simulation domain have also been explicitly described. The added text reads as follows:

To describe the electric double layer (EDL) formed at the graphene–electrolyte interface, a coupled electrostatic and ionic-transport model was implemented in the electrolyte domain. The electrolyte was modeled as a spherical domain with a radius of 4 μm in contact with the graphene surface. The electric potential and ionic concentration distributions were obtained by solving the coupled Poisson–Nernst–Planck (PNP) equations.

Boundary Conditions

At the graphene–electrolyte interface, an electrically active boundary condition was applied to account for the formation of the EDL. The interfacial electric potential was coupled to the charge accumulated in the electrolyte and the corresponding EDL capacitance. This boundary condition enables the applied electrostatic potential to generate the ionic redistribution required for the formation of the diffuse layer adjacent to the graphene surface.

At the outer boundary of the spherical electrolyte domain, the electric potential was fixed at

$$\phi = 0,$$

which represents the bulk-electrolyte reference potential. The ionic concentrations at this boundary were set to their corresponding bulk concentrations, thereby representing an electroneutral bulk electrolyte sufficiently far from the graphene surface.

The β2-microglobulin concentration at the outer boundary was prescribed according to the concentration considered in each simulation. No-flux conditions were applied where appropriate to prevent artificial loss or generation of ionic species through boundaries that do not participate in transport.

β2-microglobulin was treated as a charged species within the electrolyte. Its valence was taken as $z = -2$, based on molecular-dynamics results reported by Brancolini and co-workers. The diffusion coefficient of β2M was taken as $D = 0.0135\ \mathring{\mathrm{A}}^2/\mathrm{ps}$for aqueous solution at $T = 300\mathrm{K}$ and pH = 7.

The computational domain and boundary conditions were selected such that the outer boundary is sufficiently far from the graphene surface compared with the characteristic EDL length scale. Consequently, the outer boundary approximates bulk-electrolyte conditions and minimizes its influence on the calculated interfacial potential and concentration profiles.

The EDL capacitance was calculated using the Gouy–Chapman formulation. In the revised model, the valence term in the Gouy–Chapman capacitance expression was carefully checked. A typographical error in the previous version, in which the valence $z$was incorrectly written as 2, has been corrected in the revised equation. The corrected formulation is used consistently throughout the calculations.

These boundary conditions and material parameters provide the basis for calculating the spatial distributions of electrostatic potential and ionic concentration near the graphene–electrolyte interface and, consequently, the concentration-dependent modification of the graphene electronic properties.

[54]- Mouro, João, et al. "Analytical modeling and experimental characterization of drift in electrolyte-gated graphene field-effect transistors." *npj 2D Materials and Applications* 9.1 (2025):
